# Gene regulation in *Kluyveromyces marxianus* in the context of chromosomes

**DOI:** 10.1371/journal.pone.0190913

**Published:** 2018-01-18

**Authors:** Du Toit W. P. Schabort, Stephanus G. Kilian, James C. du Preez

**Affiliations:** Department of Microbial, Biochemical and Food Biotechnology, University of the Free State, Bloemfontein, South Africa; Tulane University Health Sciences Center, UNITED STATES

## Abstract

Eukaryotes, including the unicellular eukaryotes such as yeasts, employ multiple levels of gene regulation. Regulation of chromatin structure through chromatin compaction cascades, and influenced by transcriptional insulators, might play a role in the coordinated regulation of genes situated at adjacent loci and expressed as a co-regulated cluster. Subtelomeric gene silencing, which has previously been described in the yeast *Saccharomyces cerevisiae*, is an example of this phenomenon. Transcription from a common regulatory element located around a shared intergenic region is another factor that could coordinate the transcription of genes at adjacent loci. Additionally, the presence of DNA binding sites for the same transcription factor may coordinate expression of multiple genes. Yeasts such as the industrially important *Kluyveromyces marxianus* may also display these modes of regulation, but this has not been explored to date. An exploration was done using a complete genome and RNA-seq data from a previous study of the transcriptional response to glucose or xylose as the carbon source in a defined culture medium, and investigating whether the species displays clusters of co-localised differentially expressed genes. Regions of possible subtelomeric silencing were evident, but were non-responsive to the carbon sources tested here. Additionally, glucose or xylose responsive clusters were discovered far from telomeres which contained some of the most significantly differentially expressed genes, encoding enzymes involved in the utilisation of alternative carbon sources such as the industrially important inulinase gene INU1. These clusters contained putative binding sites for the carbon source responsive transcription factors Mig1 and Adr1. Additionally, we investigated the potential contribution of common intergenic regions in co-regulation. Some observations were also made in terms of the evolutionary conservation of these clusters among yeast species and the presence of potential transcriptional insulators at the periphery of these clusters.

## Introduction

It is known that in *Saccharomyces cerevisiae* multiple levels of gene regulation exist. Chromatin silencing and desilencing occurs in regions on the genome especially close to the telomeres, known as X and Y elements, which are enriched in transcription factor binding sites [1]. These regions are located approximately six to seven kilobases from the telomeres [1] and the process is, therefore, named subtelomeric gene silencing. Chromatin silencing spreads from these regions and may silence several genes together. The physical organisation of the genes at neighbouring loci might be a mechanism to coordinate expression of genes with similar functions. It has also been shown that, contrary to expectation, tRNA genes could serve as transcriptional insulators [2]. These regions on the genome associate via TFIIIC, the RNAP III general transcription factor complex, to the nuclear matrix or nuclear cytoskeleton, thereby organising chromatin into functional units and isolating the transcriptional unit from the effects of neighbouring regulatory elements and gene silencing [2]. Also, transcription factors binding in a shared intergenic region between two genes with an outwardly oriented transcriptional direction as a reverse forward direction pair (RF), may allow co-regulation of the two target genes. This mode, which is referred to here as outward transcription, is related to, but somewhat distinct from, divergent transcription from bidirectional promoters. It can also involve the sharing of a common regulatory element, but is associated with non-coding (anti-sense) transcript formation [3, 4]. Finally, evolutionary events such as gene duplication would create a copy also of the gene regulatory elements, along with their associated genes. This might be expected to result in co-regulation of such a duplicated region.

It is reasonable to believe that genes with common functions and which are associated with the same metabolic pathways would be co-regulated through a combination of these mechanisms. For instance, by co-localisation on the genome, they may employ more than one of the above-mentioned higher level regulatory features. By considering gene expression profiles and transcriptional orientation in the context of chromosomes, such patterns may be revealed [1]. A comparison of the conservation of gene order among multiple sister species may strengthen the notion that the ordering and proximity of a set of genes improves the functioning of a gene cluster across species [5]. Some transcription factors bind many regulatory regions and are responsible for distinct metabolic or lifestyle programs. Mig1 and Adr1 are two important transcription factors involved in glucose repression and derepression in *S*. *cerevisiae* [6]. Mig1, a zinc finger protein that recognises a conserved GC box described as [GC][CT]GGGG, is a transcriptional repressor in *S*. *cerevisiae* [7, 8]. It was later shown that a flanking AT box was also important in *S*. *cerevisiae*, thus the motif may be better described as [ATG][AT][AT][AT][ATG]N[GC][CT]GGGG [9]. Adr1 is an activator of many genes involved in the utilisation of alternative carbon sources by *S*. *cerevisiae*, especially those encoding enzymes involved in peroxisomal β-oxidation [10]. The consensus pattern for Adr1 has been described as [TGA][TC]GG[AG]G [11]. It usually binds as a dimer in opposite directions between two and 36 bp apart, and the more precise motif can be thus be described as C[CT]CC[GA][TCA]N{2–36}[TGA][TC]GG[AG]G, the reverse being identical. The longer, more precise patterns make computational identification of such binding sites more accurate and renders the chances of random occurrence of matching sequences less likely by orders of magnitude.

The yeast *Kluyveromyces marxianus* has in recent years gained increasing attention as a potential biofuel producer with several advantages over *S*. *cerevisiae*, such as a high growth rate, thermotolerance and the ability to naturally utilise pentoses such as xylose [12, 13]. The transcriptomic response of *K*. *marxianus* strain DMKU3-1042 to glucose and xylose in a complex culture medium has been explored by Lertwattanasakul et al. [14]. More recently, we performed an RNA-seq analysis combined with a multi-network analysis of *K*. *marxianus* UFS-Y2791 grown in a chemically defined medium, also with glucose and xylose as respective carbon substrates [15]. A strong up-regulation of peroxisomal metabolism in a xylose medium in the absence of glucose was evident, as well as up-regulation of several genes involved in the utilisation of alternative carbon sources, including genes of the 2-methylcitrate cycle. Based on the enumerative method of heptamer frequency comparison, it was found that, firstly, Adr1 and, secondly, Mig1 were strong candidates as major regulators of up-regulated genes in *K*. *marxianus* in a xylose medium containing no glucose, thus representing glucose derepressed conditions [16]. RNA-seq data of the Adr1 and Mig1 transcripts also supported their respective roles as activator and repressor of transcription. *K*. *marxianus* produces the industrially important enzyme inulinase, and it was shown earlier by Lertwattanasakul et al. [17] that two putative binding sites for Mig1 existed in the regulatory region of the INU1 gene of *K*. *marxianus* DMKU3-1042 that are perfectly conserved in four other strains of this species. These sequences were TTAAATCCGGGG at bp 155 and TTTTTCCTGGGG at 500 bp from the translation start site, respectively. Both match the combined AT box, GC-box consensus as previously described by Lundin et al. [9].

It was also found that the RNA-seq data from *K*. *marxianus* UFS-Y2791 could be effectively mapped to a complete genome of *K*. *marxianus* DMKU3-1042 [16]. This made it possible to consider differential gene expression in the context of chromosomes. In this report, RNA-seq data were mapped to complete chromosomes to discover the presence of clusters of co-localised genes that are also co-regulated. The potential roles of chromatin silencing, outward transcription and transcriptional insulators are all considered. Further, the functional organisation of the genome in terms of metabolic pathways that were up-regulated in the xylose medium compared to the glucose medium was investigated. These were the up-regulated genes of peroxisomal metabolism, the 2-methylcitrate cycle and sugar transporters. Also, transcription factor motif searches were done to determine whether putative binding sites for Adr1 and Mig1 might co-localise with any up-regulated gene clusters.

## Materials and methods

Experimental protocols were described in Schabort el al. [15] and are summarised below.

### Strains and cultivation

*K*. *marxianus* UFS-2791 was cultivated in aerobic shake flasks at 35°C in a chemically defined medium containing glucose or xylose as carbon substrate. RNA was extracted in mid-exponential phase [15].

### RNA-seq and differential expression

RNA-seq reads from *K*. *marxianus* UFS-Y2791 from previous work [15] were mapped to the complete genome of *K*. *marxianus* DMKU3-1042 [14] using TopHat2 [18, 19] in Galaxy [20]. The genome annotation file (.gff3) for strain DMKU3-1042 was used for the analysis to serve as quantitation window for differential expression testing in CuffDiff [21]. Alignment pile-ups were converted to intervals using the Pileup-to-Interval tool in SAM Tools [22] as implemented in Galaxy. Throughout, up-regulation refers to genes that were up-regulated in the xylose medium compared to the glucose medium.

### Enrichment for clusters of concordantly transcribed genes

An algorithm was developed for *Reactomica* [15], implemented in the Wolfram language for mapping differential expression to chromosomes and to map intervals from alignment pile-ups to DNA for visualisation. To find segments of concordantly transcribed genes indicative of chromosomal level regulation (differentially regulated in the same direction), the number of consecutive genes considered as a segment was first decided on. Setting the segment length cut-off too low would result in too little evidence for concordant transcription, while setting the length cut-off too high might exclude regions of true chromosomal level regulation but without being activated in either state by transcription factors (most genes are constitutively transcribed). Accordingly, a segment length of six consecutive genes was chosen, and to allow for the additional level of transcription factor regulation, a minimum of four genes in a segment had to be concordantly transcribed with the rest constitutively transcribed. To estimate the expected probability of such segments, a randomised sampling method was used. During each of 100 000 iterations, the ordered gene lists per chromosomes were joined, this gene list shuffled, the expression states of six sequential genes sampled, and the fraction of segments with at least four concordantly transcribed genes ((U> = 4 and D = = 0) or (D> = 4 and U = = 0)) was obtained (where U and D denote up and down regulation respectively, in xylose medium compared to glucose medium and C denote constitutive regulation). After each iteration, this concordant fraction was compared to the concordant fraction in the single observed genome, which was analysed as separate chromosomal gene lists. The fraction of occurrences in which the randomised segments displayed a higher concordant fraction than the observed genome was used as the p-value for the hypothesis test that the ordering of gene expression was random. Code for random sampling and discrete statistics was developed in Python. (Also see S1 Stats Report for a discussion on the statistical methodology.)

### The association between concordant transcription and a common intergenic region

A test was performed to see whether it was more likely for a pair of genes that share an intergenic region to display concordant transcription (regulated in the same direction), as compared to those pairs with different intergenic regions. Genes located adjacent on the chromosome were assigned to pairs and pairs were classified according to their pattern of differential expression as combinations of up (U), down (D) and constitutive (O), and subsequently as concordant (UU+DD) and discordant (UO+DO+UD) transcription. The pairs with both genes constitutively transcribed (OO) were excluded from the analysis as they served no purpose in classification. In total, 1 041 out of the 5 209 pairs analysed were classified as concordant or discordant. The likelihood ratio, *L*, was calculated as the ratio of probability values as described below, in which the symbol N refers to the number of pairs with a certain orientation of transcription.

$$L=\frac{P(concordant|common)}{P(concordant|different)}$$

$$P(concordant|common)=\frac{N(concordant|common)}{N(concordant|common)+N(discordant|common)}$$

$$P(concordant|different)=\frac{N(concordant|different)}{N(concordant|different)+N(discordant|different)}$$

As a significance test, the R implementation of Fischer’s exact test [23] was used to compare the groups of pairs with common and different transcriptional orientations as described in the contingency Table 1 below, resulting in an odds ratio and a p-value for the odds ratio not being equal to unity.

**Table 1:** Contingency table for Fischer’s exact test [23] used in this study.

	common (RF)	different (FF,RR, FR)
concordant	24	49
discordant	245	723

### Motif searches

The dimeric binding model of Adr1 binding used was similar to that of Cheng et al. [11] as a regular expression C[CT]CC[GA][TCA]N{2–36}[TGA][TC]GG[AG]G, the reverse being identical. The core recognition site (GC-box) for Mig1 was described as [GC][TC]GG[GA]G (and C[CT]CC[AG][GC] in the opposite direction) [8]. The more restrictive GC box, AT box pattern was modelled as [ATG][AT][AT][AT][ATG]N[GC][TC]GGGG and the reverse of this pattern as CCCC[GA][GC]N[TAC][AT][AT][AT][CAT], based on the binding sequences reported by Lundin et al. [9] or as [TC][AG]CACCC[AG] from the *Saccharomyces* Genome Database (SGD) [24]. The Adr1 DNA binding motif was modelled either as [TGA][TC]GG[AG]G as previously described [11], or as a dimeric binding site C[CT]CC[GA][TCA]N{2–36}[TGA][TC]GG[AG]G. The DNA binding motif for Aft1 was modelled as [TC]GCACC[TC] from de Freitas at al. [25].

## Results

**Fig 1:**
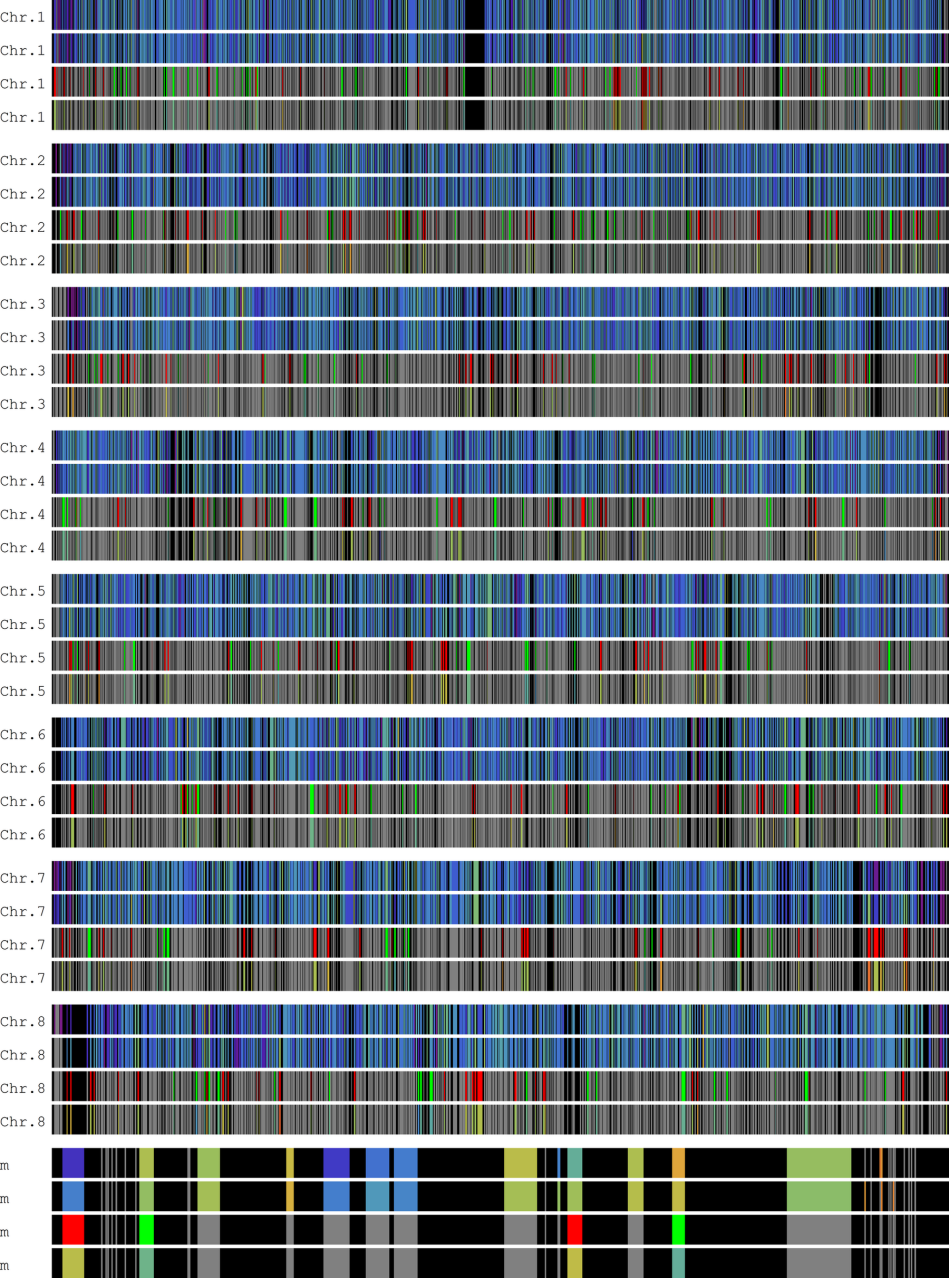
Visual representation of genomic chromosomes 1 to 8 and the mitochondrial chromosome mapped with RNA-seq data from *K*. *marxianus* UFS-Y2791 using the genome annotation by Lertwattanassakul et al. [14]. Black represents intergenic regions. Track 1, normalised transcript levels with glucose as carbon source; Track 2, normalised transcript levels with xylose as carbon source. Warmer colours represent highly expressed genes with red indicating the highest; colder colours represent lowly expressed genes with violet indicating the lowest. Track 3, up/down classifier scheme (as xylose/glucose): red, up-regulated; green, down-regulated; grey, constitutively expressed. Track 4, colouring scheme reflects the log2 of fold changes, from glucose to xylose (as xylose/glucose): warmer colours represent the highest positive fold changes, colder colours the highest negative fold changes and grey the constitutively expressed genes. Also see Table A in S1 Text for transcript levels and fold changes, and Fig J in S1 Text for a colour key.

Out of a total of 5 162 genes, 323 were up-regulated in the xylose medium, while 245 were down-regulated in RNA-seq data of *K*. *marxianus* UFS-Y2791 [15]. Differential expression values were mapped to the complete chromosomes of *K*. *marxianus* DMKU3-1042 as annotated by Lertwattanassakul et al. [14] and represented in Fig 1 (also see Table A and Fig J in S1 Text). In all chromosomes, except for chromosomes 5 and 6, there seems to be a pattern in that several genes close to the telomeres have low expression levels under both conditions of glucose or xylose as carbon source (Fig 1, tracks 1 and 2). These resemble a reasonable positioning for subtelomeric silencing. Notably, these regions were non-responsive to the provision of glucose or xylose as carbon source. However, further away from telomeres, other gene clusters that displayed concordant transcription as either up-regulated or down-regulated sets were visible on the chromosomes. The p-value for the hypothesis test for random occurrence of at least four concordantly transcribed genes in a segment of six adjacent genes ((U> = 4 and D = = 0) or (D> = 4 and U = = 0)) was calculated at 1.5×10^−4^, indicating that this configuration is highly unlikely to occur by chance.

**Fig 2:**
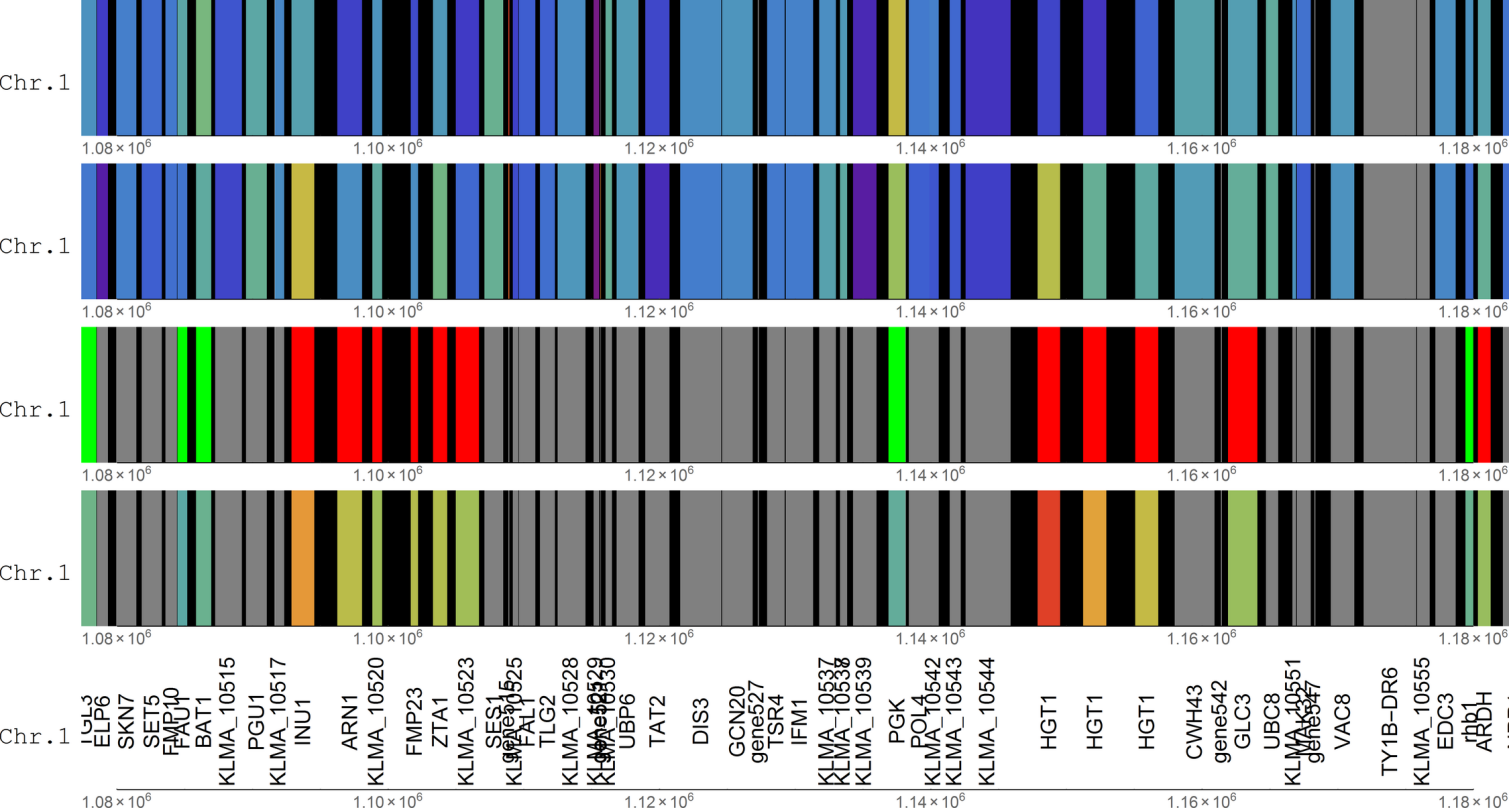
Visual representation of two clusters of up-regulated genes on chromosome 1. See Fig 1 for explanation of the colouring of the tracks. Gene515 is a putative tRNA gene immediately downstream of the cluster containing INU1, downstream of SES1. Gene542 is another putative tRNA gene in the HGT1 cluster, immediately downstream of the HGT1 repeat. Immediately downstream of the HGT1 cluster is tRNA Gene547, and halfway between the two clusters, tRNA Gene527 is visible. Also see Table A in S1 Text for transcript levels and fold changes, and Fig J in S1 Text for a colour key.

On initial inspection, two such gene clusters immediately visible on chromosome 1 were those between 1 Mbp and 1.2 Mbp, which were both up-regulated in the xylose medium compared to the glucose medium, in a surrounding setting of constitutively expressed genes (Fig 2 and Fig A in S1 Text). Another was visible on chromosome 7, between at 874 Kbp. These three regions were classified as having at least four concordantly transcribed genes regulated in the upward direction in xylose medium, and hence a p-value of occurrence by change of 1.5×10^−4^.

The first gene cluster contained the inulinase gene INU1 on the 5’ end, followed by ARN1 (siderophore iron transporter ARN1), KLMA_10520 (uncharacterised protein AN0679), FMP23 (protein FMP23), ZTA1 (probable quinone oxidoreductase) and KLMA_10523 (uncharacterised protein) (Fig 2). This cluster had all six genes up-regulated. The second gene cluster contained three repeats of the putative high-affinity glucose transporter HGT1 as well as GLC3, with four genes up-regulated. These three paralogs likely originated via gene duplication. In both of these clusters it seems that the gene on one end of the cluster was the most significantly up-regulated, with a decreased up-regulation further away from that side. The fold change in INU1 transcript level was 90.4-fold, decreasing to 3.2-fold for gene KLMA_10523 on the opposite side of the gene cluster. In the second cluster, the three copies of HGT1 showed respective fold-changes of 843, 61 and 12-fold, while the GLC3 gene showed a fold change of 2.3. This pattern bears a resemblance to the mechanism of chromatin silencing which spreads from one locus to other loci [1]. However, the highest fold changes within the clusters were on the centromeric side, and the regions were far from the telomeres. Notably, in both the clusters, a putative tRNA gene occurs on the periphery (Fig 2) which might serve as transcriptional insulator [2]. Another putative tRNA was found downstream of the HGT1 cluster, while another was present halfway between the two clusters.

### Biological significance of gene clustering

In the three regions considered to be significant, 14 genes were found to be up-regulated. This is a small percentage (14.3%) out of the total of 323 up-regulated genes on the whole genome. However, these contain some of the genes unique to *K*. *marxianus* which are also of industrial importance, including the inulinase gene INU1, as well as sugar transporters, the FOX2 gene of peroxisomal β-oxidation, some genes of the 2-methylcitrate cycle, and others apparently involved with metal ion metabolism.

### Significance of outward transcription in co-regulation of adjacent gene pairs

A statistical test was performed to estimate whether gene pairs with a common intergenic region (RF orientation) showed a higher likelihood of being concordantly transcribed, as opposed to those pairs different intergenic regions (FF, RR, FR) (see Materials and Methods). Of 5 209 adjacent gene pairs, only those pairs with at least one differentially regulated gene were investigated (1 041), of which 269 had a common intergenic region and with 24 displaying concordant transcription, while 772 pairs had different intergenic regions of which 49 displayed concordant transcription. The likelihood ratio *L* was calculated at 1.41, suggesting that there was some bias towards concordant transcription when sharing an intergenic region, as compared to pairs with different intergenic regions. However, the Fisher’s exact test resulted in an odds ratio of 1.44, with a p-value of 0.166, suggesting that this was not a statistically significant global observation.

However, the discovery of co-regulated gene clusters based on positional enrichment of transcriptional data may evidently be explained away partly by outward transcription, since three adjacent pairs of outwardly transcribed genes form a cluster of six genes and hence up to three of the pairs may have RF orientation. Each of the three clusters investigated were initially considered. It showed that for the INU1 cluster, four out of six concordantly regulated genes shared two intergenic regions (two pairs centred around two intergenic regions), explaining 66.6% of the concordant regulation. For the FOX2 cluster, two out of the four concordantly expressed genes had a common intergenic region, explaining 50% of the concordant regulation. For the HGT1 sugar transporter cluster, none of the concordantly transcribed genes had a common intergenic region.

An estimate was also calculated to determine whether there was reason to believe that concordantly regulated clusters should arise due to a higher than expected prevalence of gene pairs with a common intergenic region (with RF orientation), regardless of the concordance of their expression patterns. Considering only those pairs with at least one differentially expressed gene (1 041 pairs), the expected number of RF pairs was 260.25 (exactly 25% of the cases), assuming randomised orientation. The observed number was 269, which is not significantly different. Hence, skewed orientation of gene pairs does not seem to contribute significantly to finding concordantly regulated clusters.

### Functional organisation, evolutionary conservation and regulatory potential of the gene cluster containing the inulinase gene INU1

**Fig 3:**
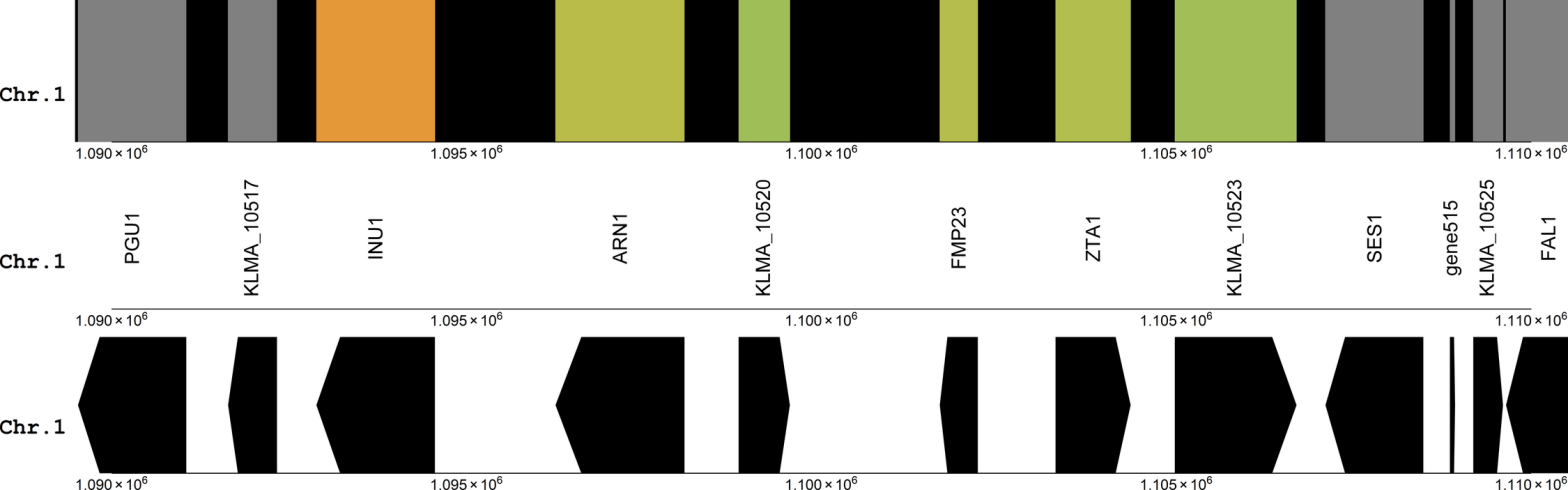
The gene cluster containing INU1, between 1.09 Mbp and 1.11 Mbp on chromosome 1, showing the direction of transcription. The colouring scheme reflects the log2 of fold changes, from glucose to xylose (as xylose/glucose): warmer colours represent the highest positive fold changes, colder colours the highest negative fold changes and grey the constitutively expressed genes. The direction of the arrows indicates the direction of transcription. Gene515 is a putative tRNA gene immediately downstream of the cluster containing INU1.

Fig 3 shows the direction of gene transcription in the gene cluster containing INU1. To gain insight into the functional organisation of the up-regulated gene cluster containing the biotechnologically important inulinase gene INU1, the functional annotation of the two uncharacterised proteins KLMA_10520 and KLMA_10523 was considered.

Gene KLMA_10520 has the GO terms "FMN binding [GO:0010181] and oxidoreductase activity [GO:0016491]" associated on the UniProt database. For gene KLMA_10523, a search on ProtoNet [26] suggested that the protein may bind to the enzyme protein phosphatase type 1, based on structural similarity with other proteins in its protein structural cluster from various species, including GIP1 in *S*. *cerevisiae*. Several highly similar sequences were found by a BLASTP search, but all of these were to uncharacterised proteins. The best characterised was the GIP1 gene product, which is a meiosis-specific regulatory subunit of the Glc7 protein phosphatase [27]. Using BLASTP, GIP1 was found but at a low statistical significance with only a short region matching GIP1. DELTA-BLAST improved the query coverage to 35% with an improved statistical significance (E-value = 2e-05) to GIP1 (see Fig B in S1 Text). The matching region was 197 bp long with a 40% similarity and 26% identity (Fig C in S1 Text). A conserved domain or protein family was not reported by DELTA-BLAST.

Perhaps most significant in assigning putative homology to the KLMA_10523 gene was its genomic context. The genomic view of the *Saccharomyces* Genome Database [28] showed that both the ZTA1 and FMP23 genes are present in *S*. *cerevisiae* as well, and transcribed in the same orientation as in *K*. *marxianus* (compare Fig 3 and Fig D in S1 Text). In both species, transcription of the genes ZTA1 and FMP23 occurs away from each other where their coding regions are separated by a short intergenic region. The organisation of this region of the genome was thus conserved after the genome duplication and reshuffling events in the evolutionary past of the Saccharomycetes. In *S*. *cerevisiae*, GIP1 is found directly downstream of ZTA1 and transcribed in the same orientation as ZTA1. This was found to be exactly the situation in *K*. *marxianus* (compare Fig 3 and Fig D in S1 Text). Most probably, GIP1 and KLMA_10523 had a common ancestor. Following the same rationale, aligning KLMA_10521 with the RPS11B protein, the ribosomal subunit gene immediately downstream from FMP23 in *S*. *cerevisiae* did not result in any significant similarity. ARN1 was found on chromosome VIII of *S*. *cerevisiae* (Fig E in S1 Text). The amino acid sequence of the EFM1 gene product, which occurs immediately upstream of ARN1 (see Fig E in S1 Text), had a closest match to the protein of the YHL039W gene in *K*. *marxianus*, namely 906 968 bp to 908 632 bp on chromosome 8 (AP012220.1), and not on chromosome 1 where the gene cluster containing INU1 is found. The closest match to the gene product of INU1 of *K*. *marxianus* in *S*. *cerevisiae* was the invertase encoded by SUC2. In *S*. *cerevisiae*, SUC2 is found on chromosome IX (Fig F in S1 Text). The Yeast Gene Order Browser [5] is a useful tool to investigate synteny among regions on yeast genomes. Fig G in S1 Text shows that GIP1, ZTA1 and FMP23 are conserved in a region of synteny in all pre-genome duplication yeasts, along with FAL1, CIS1, the tRNA gene tV-UAC, and SES1. Conversely, ARN1 is only shared among *Lanchaea thermotolerans* (*K*. *thermotolerans*), *L*. *waltii* (*K*. *waltii*) and *S*. *cerevisiae*, but absent from *K*. *lactis*, *L*. *kluyveri* (*S*. *kluyveri*) and other pre-genome duplication yeasts (Fig H in S1 Text).

In summary, it is thus likely that FMP23, ZTA1 and KLMA_10523 (GIP1) in *S*. *cerevisiae* originated from a single genomic region in the last common ancestor, and were kept intact in *S*. *cerevisiae* after the genome duplication event and subsequent extensive rearrangements, while ARN1 and KLMA_10520 were moved to other regions. The potential chromatin silencing insulator tRNA Gene515 (tV-UAC) was also conserved in this segment among the 11 yeast species investigated using the Yeast Gene Order Browser (Fig G in S1 Text).

Notably, both FMP23 and ARN1 have been implicated in the response to metal ion concentration. It has been suggested that FMP23 (YBR047W) was involved in copper or iron balance, as in *S*. *cerevisiae* it is up-regulated in response to copper depletion and its regulatory region is enriched with binding sites for the Aft1 and Aft2 transcription factors [29]. Aft1 and Aft2 control a number of iron responsive genes [29]. ARN1 also is regulated by Aft1 [30]. This raises the question whether the Aft1 and Aft2 binding sites possibly originated in the common ancestor of *Kluyveromyces* and *Saccharomyces*, which suggests that they might also be present in *K*. *marxianus* and concentrated over the gene cluster containing INU1.

**Fig 4:**
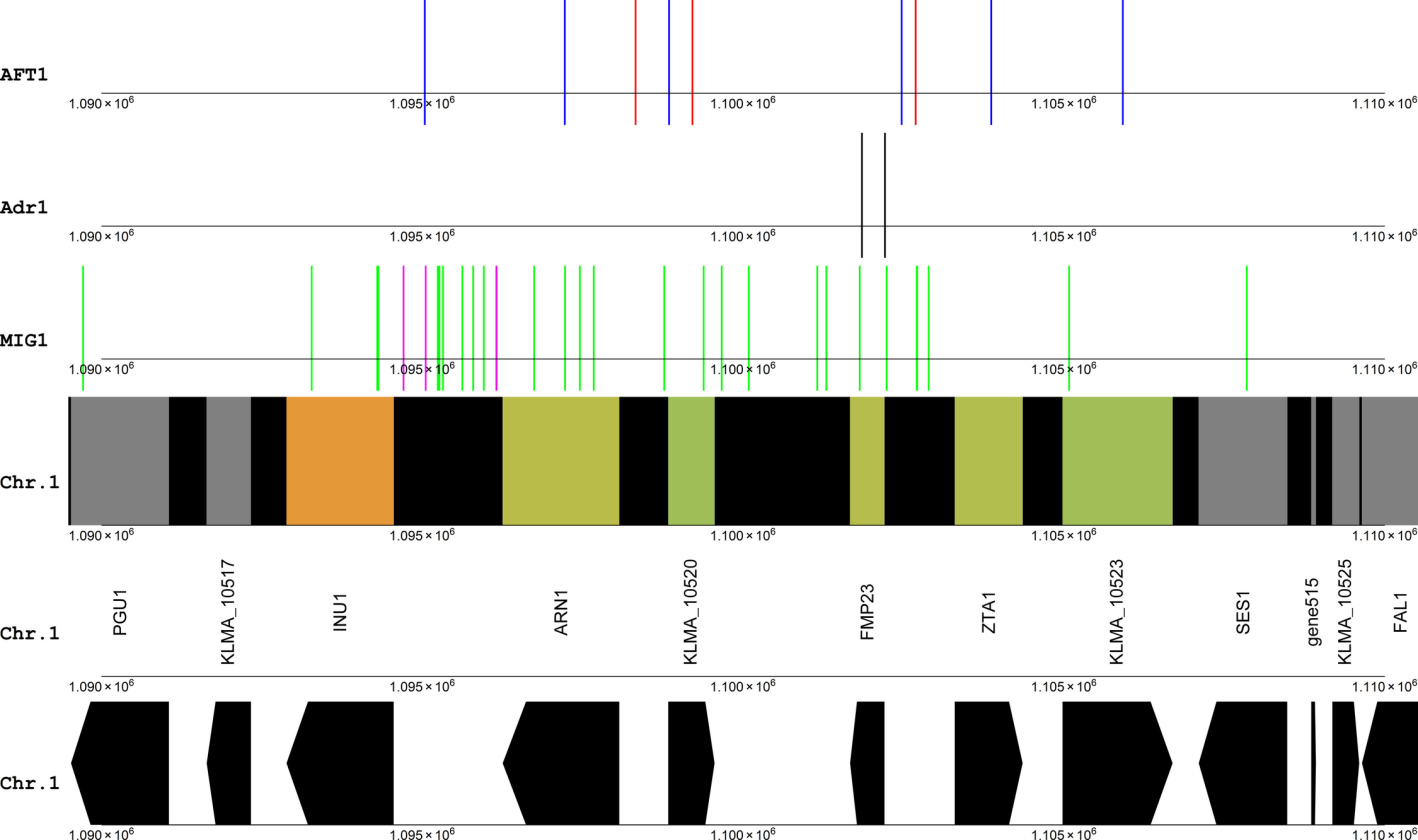
Candidate DNA binding sites for Adr1, Mig1 and Aft1/Aft2 in the vicinity of a gene cluster containing INU1, between 1.09 Mbp and 1.11 Mbp on chromosome 1. The colouring scheme reflects the log2 of fold changes, from glucose to xylose (as xylose/glucose). Black lines, candidate Adr1 binding sites using the dimeric binding model; magenta lines, candidate Mig1 binding sites using the GC box, AT box model; green lines, candidate Mig1 binding sites using the core GC box model; blue lines, Aft1/Aft2 consensus motif [TC]GCACC[TC]; red lines, Aft1/Aft2 consensus motif PyPuCACCCPu ([TC][AG]CACCC[AG]).

The exact consensus pattern GTGCACCC for the Aft1 binding site in the regulatory region of FMP23 did not result in any matches in the gene cluster investigated here. However, using the consensus motif [TC]GCACC[TC] from de Freitas at al. [25] revealed two potential binding sites for FMP23 and ZTA1, as well as one that was in the region of the core promoter of ARN1, but which may equally serve as a regulator of the KLMA_10520 gene (Fig 4). In addition, the motif was also found at the region upstream of the inulinase gene INU1. In SGD, the motif is described as "PyPuCACCCPu” ([TC][AG]CACCC[AG]), which is more specific. Using this pattern revealed that the binding site in the core promoter region of ARN1 matched the more specific pattern, as did the pattern closest to the ZTA1 start site (Fig 4, red lines). If these were true functional binding sites for Aft1, this cluster may be responsive to concentrations of metal ions. This finding might also have a direct application, as it suggested that inulinase production in this yeast might be further inducible by adjustment of the concentration of metals such as iron or copper.

Since the alleviation of glucose repression is a likely explanation for the up-regulation of many genes in *S*. *cerevisiae*, it was expected that Adr1 and Mig1 may regulate this response [18]. The short and degenerate core binding site of Adr1 resulted in too many putative binding sites to be considered. The much more restrictive dimeric binding model revealed only two binding sites in the whole region, one of which was directly upstream of the FMP23 gene (Fig 4). Mig1 is also an important transcriptional repressor in *S*. *cerevisiae*. Both the GC box motif and the combined AT box, GC box motifs were scanned through the gene cluster. Three of the combined AT box, GC box motifs were found in this region (Fig 4, magenta lines), of which two were the sites reported by Lertwattanasakul et al. [18]. The other motif is in the same upstream intergenic region of INU1, directly downstream of the ARN1 gene. A larger number of the more degenerate GC box motifs was also detected (Fig 4, green lines), which appeared to be concentrated over the up-regulated gene cluster as opposed to the neighbouring regions. The regulation of this gene cluster may thus both be responsive to the concentration of the carbon source and to metal ions.

**Fig 5:**
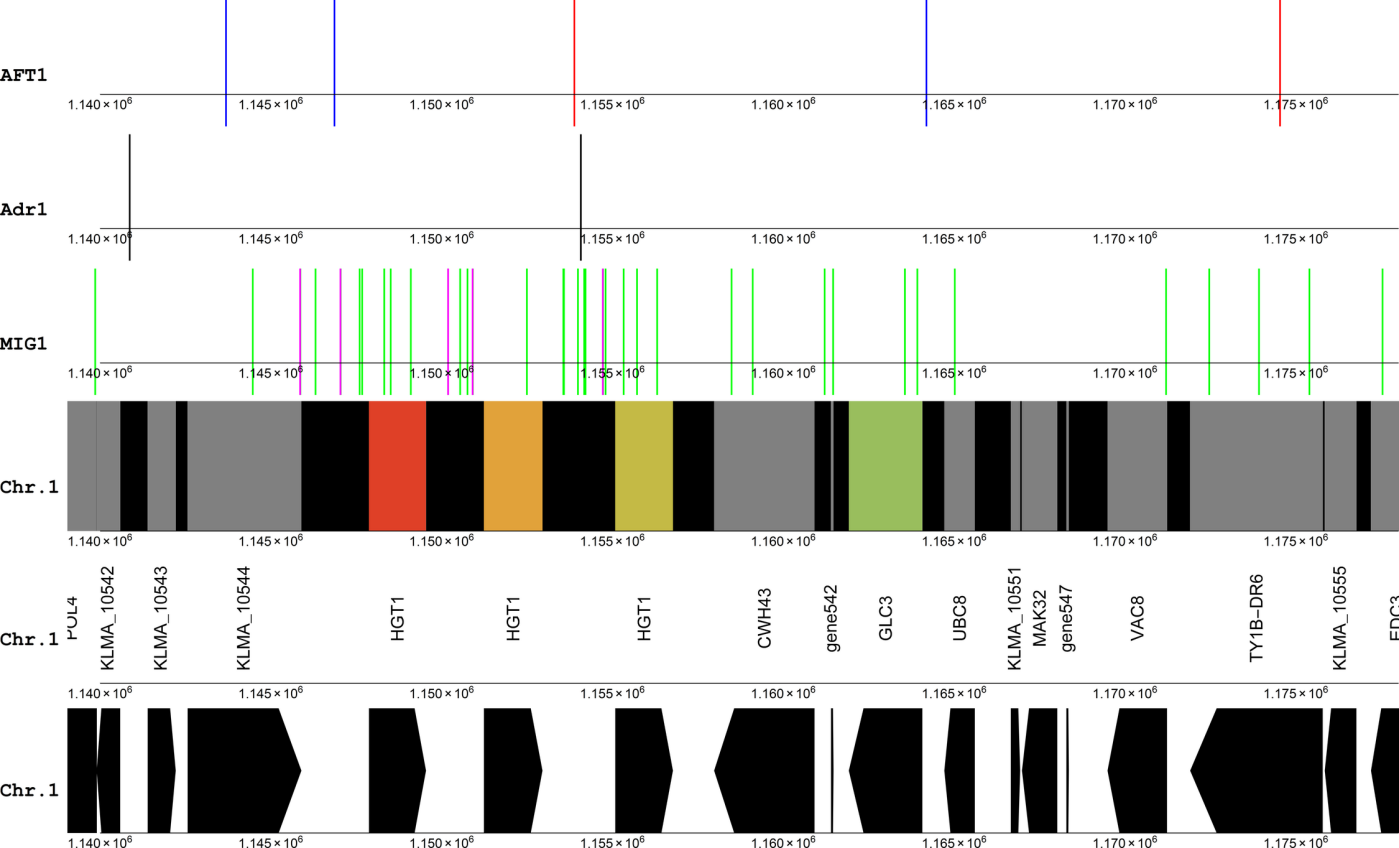
Candidate DNA binding sites for Adr1, Mig1 and Aft1/Aft2 in the vicinity of a gene cluster containing three copies of HGT1, between 1.14 Mbp and 1.17 Mbp on chromosome 1. The colouring scheme reflects the log2 of fold changes, from glucose to xylose (as xylose/glucose). Black lines, candidate Adr1 binding sites using the dimeric binding model; magenta lines, candidate Mig1 binding sites using the GC box, AT box model; green lines, candidate Mig1 binding sites using the core GC box model; blue lines, Aft1/Aft2 consensus motif [TC]GCACC[TC]; red lines, Aft1/Aft2 consensus motif PyPuCACCCPu ([TC][AG]CACCC[AG]). Gene542 is a putative tRNA gene in the HGT1 cluster, immediately downstream of the HGT1 repeat, and immediately downstream of the HGT1 cluster is tRNA Gene547.

The dimeric Adr1 binding sites were notably absent in the gene cluster containing the sugar transporter gene HGT1 (Fig 5), except for one in-between two of the HGT1 gene copies. The same HGT1 gene also had a candidate site for Aft1 binding. However, each of the three HGT1 copies had candidate binding sites for the GC box, AT box Mig1 model in their upstream regulatory regions, which were the only such sites in the whole region. As was the case with the INU1 cluster, candidate Mig1 sites were concentrated around the genes with the most significant up-regulation. Since gene duplication is the most likely explanation for observing three copies of the same gene, it is expected that the same regulatory features were also copied and may have been conserved. In terms of the positioning of Mig1, Adr1 and Aft1, the three upstream regulatory regions do not bear a resemblance with one another.

### Genes of the 2-methylcitrate cycle are up-regulated and co-localised

**Fig 6:**
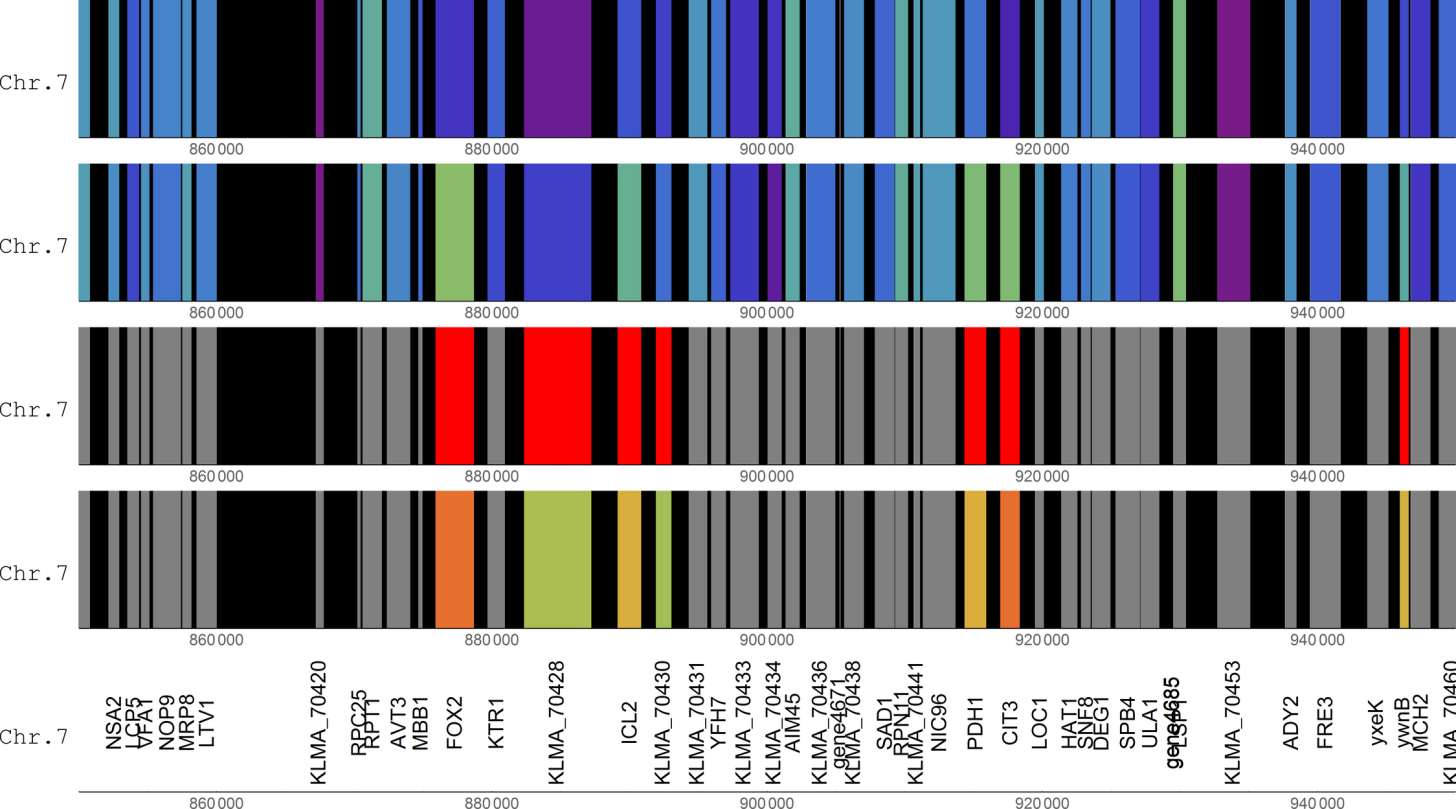
A cluster of up-regulated genes on chromosome 7. See Fig 1 for explanation of the colouring of the tracks.

A gene cluster with four up-regulated genes (FOX2, KLMA_70428, ICL2 and KLMA_70430) was found on chromosome 7 (Fig 6). The FOX2 gene encodes one of the enzymes of β-oxidation, a pathway which was strongly up-regulated in the xylose medium along with many other peroxisomal genes [14, 15]. No other peroxisomal genes were, however, found close to the FOX2 gene. Further investigation showed that peroxisomal genes were distributed among chromosomes. The up-regulated isocitrate lyase 2 gene, ICL2, was also found within the cluster. ICL2 does not encode the glyoxylate cycle enzyme Icl1, but instead encodes an isocitrate lyase isozyme belonging to the 2-methylcitrate cycle. All three genes encoding enzymes of the 2-methylcitrate cycle were strongly up-regulated in the xylose medium [15]. Strikingly, the other two genes of the 2-methylcitrate cycle, namely 2-methylcitrate dehydratase (PDH1) and citrate synthase 3 (CIT3), were found to be located close to the FOX2-containing gene cluster and adjacent to each other (Fig 6).

**Fig 7:**
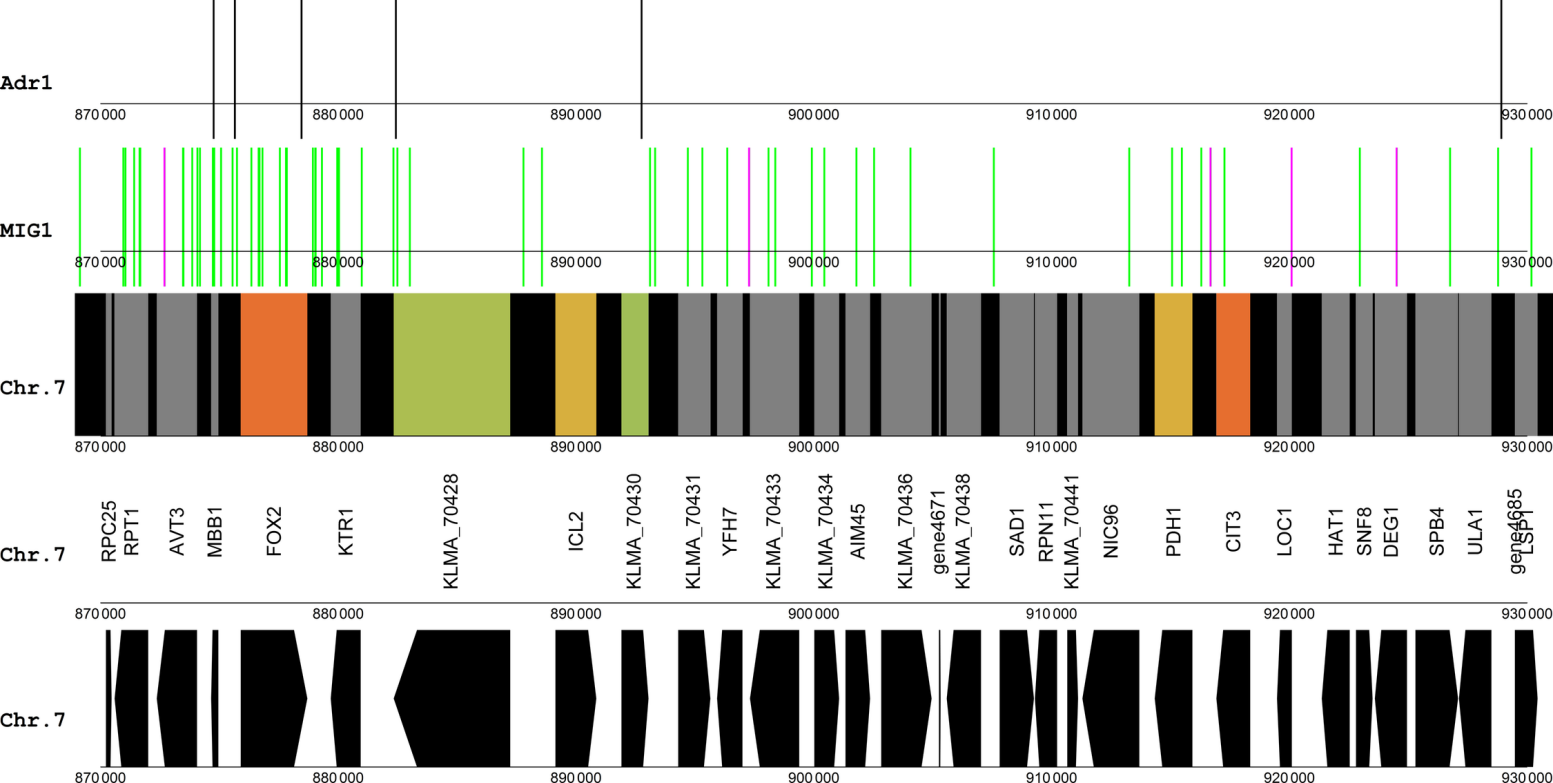
Candidate DNA binding sites for Adr1 and Mig1 near a gene cluster on chromosome 7 containing FOX2. The colouring scheme reflects the log2 of fold changes, from glucose to xylose (as xylose/glucose). Black lines, candidate Adr1 binding sites using the dimeric binding model; magenta lines, candidate Mig1 binding sites using the GC box, AT box model; green lines, candidate Mig1 binding sites using the core GC box model.

Using the dimeric binding model for Adr1 revealed one candidate binding site upstream of FOX2 (Fig 7), which is in accordance with data for *S*. *cerevisiae* where FOX2 was shown to be under the regulation of Adr1 [10]. Adr1 was also found to be the most likely candidate regulator of up-regulated genes based on the enumerative method of heptamer frequency comparisons [17]. Four other candidate Adr1 sites were found in proximity of the gene cluster, but were either inside the coding regions or downstream of the genes. The GC box, AT box motif for Mig1 resulted in one candidate binding site upstream of the PDH1 gene, immediately downstream of CIT3.

## Discussion

In this work, regions were found close to telomeres that harboured sequences of genes that were expressed at very low levels under conditions with glucose or xylose as sole carbon sources, suggesting that subtelomeric silencing might only appear under certain conditions, or that both glucose and xylose lead to silencing. Other differentially regulated gene clusters were discovered that may be under the control of chromatin desilencing in response to the carbon source. These resembled the expression patterns of subtelomeric regions found in *S*. *cerevisiae* [1], but were not located close to telomeres. The silencing of genes due to chromatin compaction and the reverse process occur in a cascaded fashion which spreads across parts of a chromosome. It seems that the most significantly up-regulated genes in these clusters were along the periphery of the clusters, with a gradual decrease in differential expression towards the other side. Notably, the two clusters investigated on chromosome 1 both had putative tRNA genes on the downstream side. The tV-UAC tRNA gene (Gene515 in *K*. *marxianus* UFS-Y2791) was found downstream from the proposed INU1-containing gene cluster (Fig G in S1 Text), which also has been conserved at this locus among the 11 of the yeast species investigated. It is noteworthy that in *S*. *cerevisiae* the tV-UAC tRNA gene is the last of a segment of six genes conserved among yeast species. Similarly, the putative tRNA tK-CUU (gene 542) is located downstream of the proposed HGT1-containing gene cluster (Fig I in S1 Text). These tRNA genes may, therefore, possibly serve as transcriptional insulators, encapsulating the genomic regions into functional clusters in three-dimensional space, tethered to the nuclear matrix [2], which could result in concordant transcriptional regulation. However, the clusters that were investigated in this study have been re-constituted from different parts of the common ancestor (Figs G-I in S1 Text). Hence, the clusters discovered in this manner of transcriptional profiling positional enrichment do not seem to be supported by syntenic mapping of gene order, although it is evident that segments within these clusters were conserved, such as the FMP23, ZTA1 and KLMA_10523 (GIP1), SES1, tV-UAC sections of the INU1 containing cluster, and the UBC8, GLC3, tK-CUU segment in the HGT1 containing cluster. These were joined to segments containing sugar utilisation genes such as INU1, which is unique to *K*. *marxianus*.

Outward transcription from common intergenic regions and regulatory elements may be a contributor to co-regulation, although the Fischer’s exact test could not conclusively provide evidence for this bias towards concordant transcription around RF oriented gene pairs. This itself might bias the discovery of gene clusters by positional enrichment using transcriptional data, partly explaining away the potential role of chromatin silencing, e.g. for the INU1 containing cluster (66%) and for the HGT1 containing cluster (50%), but not for the FOX2 containing cluster (0%).

Glucose repression involves transcription factors, where Mig1 serves as a repressor and Adr1 as an activator. These were also highlighted in previous work to be likely important in the differential response to glucose and xylose in *K*. *marxianus* [17]. The mechanism of Mig1 repression is to recruit chromatin silencing proteins such as Tup1 [31] leading to the silencing of chromatin. Thus, the exact location of Mig1 sites, such as the distance from the transcription start site and the orientation of its binding, may be less important as opposed to those transcription factors that interact with RNAPII via the Mediator complex. Candidate GC box, AT box Mig1 sites were found in the regulatory regions of these clusters, and their abundance also appeared to correlate with the most significantly up-regulated genes, notably for the INU1 containing cluster, the three HGT1 copy cluster and for the PDH1 gene. For the HGT1 gene cluster, it is expected that the gene duplication events may have resulted in co-regulation of these three genes due to copied regulatory elements. Even though the positioning of regulatory motifs for Mig1, Adr1 and Aft1 did not follow the same pattern in the three genes, it does not rule out that some of these elements might have originated from the same ancestral motifs. Whether other regulatory elements were conserved in these regions can be addressed by phylogenetic comparisons of sister species and is under investigation.

It was interesting to note that the highly up-regulated FOX2 gene of peroxisomal β-oxidation was found in a cluster together with the ICL2 gene of the up-regulated 2-methylcitrate cycle, and that the other two genes of this pathway were found to be up-regulated and located close to this cluster and adjacent to each other. Adr1, which is known to regulate peroxisomal genes, was found to have a candidate site in the regulatory region of the FOX2 gene. It was also noteworthy that the INU1 gene encoding inulinase was found in a cluster of other up-regulated genes known to be associated with the response to metal ion concentrations in *S*. *cerevisiae*. Since this segment of the genome has been conserved in *S*. *cerevisiae* even after severe reshuffling of the genome in the evolutionary past, it is speculated that the same regulatory mechanisms originated in the earlier common ancestor. In accordance with this notion, candidate binding sites for the metal responsive Aft1/Aft2 binding sites were found for these genes, as they also occur in *S*. *cerevisiae*.

## Conclusions

The genomic context of complete chromosomes provided another route of exploration of RNA-seq data in the differential expression response of *K*. *marxianus* UFS-Y2791 to glucose and xylose as respective carbon sources. The up-regulated gene clusters identified in this work, based on positional enrichment, presented an interesting perspective on gene regulation. Gene regulation of pathways such as the 2-methylcitrate cycle may be coordinated both by transcription factors and their localisation along the chromosome, in which a spread of chromatin silencing or desilencing could coordinate the regulation of a gene cluster. Outward transcription seems to be a significant contributing factor to co-regulation of the INU1 and HGT1 containing clusters. Yet for others like the genes encoding peroxisomal β-oxidation, the coordination may be more dependent on common transcription factor binding sites. Finding co-localisation of the INU1 gene with metal-responsive genes, as well as candidate binding sites for a metal responsive transcription factor, provide an interesting perspective on the use of co-regulated gene clusters. Perhaps such knowledge could be used in an industrial scenario where the adjustment of metal ion concentrations, in particular copper and iron, may be used to further improve the production of inulinase, along with a non-fermentable carbon source to impose glucose derepression. Further experimental validation of predictions would improve this line of research. Insertion of a gene along with its promoter for which its activation condition is well defined into these clusters would further support higher level gene regulation, if it adopted that of the clusters. Ultimately, additional high-throughput data in the form of chromatin immunoprecipitation should be invaluable as complementary datasets to the computational analysis of genomic and RNA-seq data presented here that suggest higher level gene regulation in this extraordinary yeast species.

## Supporting information

S1 Text Supplementary figures and tables. (PDF)

S1 Stats Report Discussion of rationale in statistical methods. (PDF)
