# Supplementary material for: Gene regulation in Kluyveromyces marxianus in the context of chromosomes
Source: PLoS One. 2018 Jan 18;13(1):e0190913. doi: 10.1371/journal.pone.0190913 (PMC5773181; doi:10.1371/journal.pone.0190913)
Supplement: S1 Text — (PDF) [file pone.0190913.s001.pdf]

Supplementary Text 1 to 'Gene regulation in *Kluyveromyces marxianus* in the context of chromosomes'

Du Toit WP Schabort*, Stephanus G Kilian and James C du Preez

Department of Microbial, Biochemical and Food Biotechnology, University of the Free State, Bloemfontein, South Africa

*For correspondence: schabortdwp@ufs.ac.za

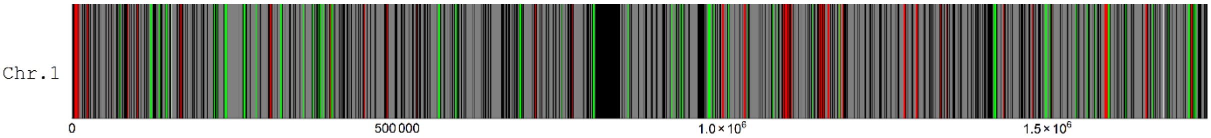

Fig 1. Visual representation of chromosome 1 mapped with RNA-seq data from *K. marxianus* UFS-2791. Notice the two clusters of up-regulated genes between 1.1 Mb and 1.2 Mb. Red, up-regulated; Green, down-regulated; Grey, constitutively expressed; Black, intergenic region.

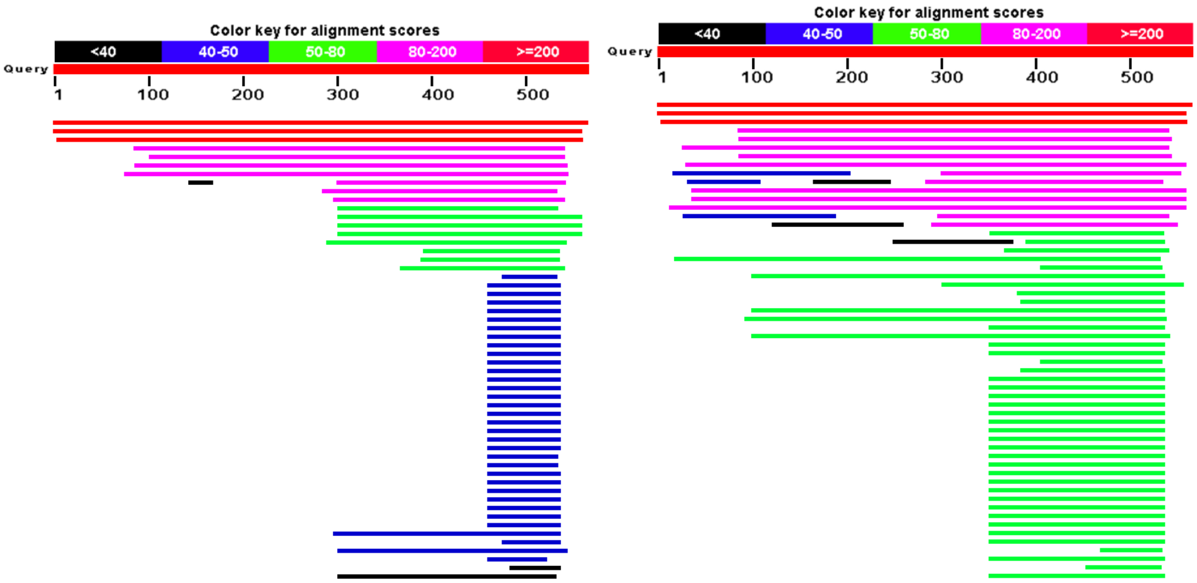

Fig 2. BLASTP and DELTA-BLAST matches to the KLMA_10523 amino acid sequence. A: GIP1 matched to 13% of the query (KLMA_10523) with an E-value of 0.006. B: GIP1 matched to 35% of the query (KLMA_10523), with an E-value of 2e-05.

```
Gip1p [Saccharomyces cerevisiae YJM1386]
Sequence ID: gb|AJQ14203.1|  Length: 639  Number of Matches: 1

Range 1: 430 to 586 GenPept Graphics                    ▼ Next Match  ▲ Previous Match
 Score            Expect  Method                       Identities     Positives      Gaps
 54.7 bits(130)   2e-05   Composition-based stats.     52/197(26%)    79/197(40%)    49/197(24%)

Query  354  DNEERSISSLEEYHMAENFSIRSKGFVQTGSIEHDQERE------RNLANMEVE---KGD  404
            DN    I S ++     NF  R+K F     +    D E E       N++ M+ +    K
Sbjct  430  DNLTHLIKSHQKKKRCVNF--RNKRFYDAFNPYVDNEEEAELSDSENISEMDTDLCIKDR  487

Query  405  KGDTVKFNKFSYLVIYDASKKCHYSESTDKLTLKIPHDGGSQTRAKIAVSALVNTETSLN  464
               +V+F++ S L+IY  SKK                                  LN
Sbjct  488  STSSVRFDENSRLLIYKKSKK----------------------------------LN  510

Query  465  IEAESTPYSDSMRLKSILKRRTNEQESIEAQRARKCDEIDASDFLEFVENHENKRRSGED  524
              +    + YS ++  +SILK + N Q    E+QRA KCD +   + FL + +  E KR+  E
Sbjct  511  KDETQSGYS-TIETRSILKTKMNSQHDEESQRASKCDTVGVAQFLHYFQYTEYKRQRNEA  569

Query  525  ILVLARERQLKNYYDDQ  541
                 R  QL  YY ++
Sbjct  570  ENYRLRGEQLSKYYSEE  586
```

Fig 3. Alignment of the KLMA_10523 amino acid sequence to that of GIP1 using DELTA-BLAST.

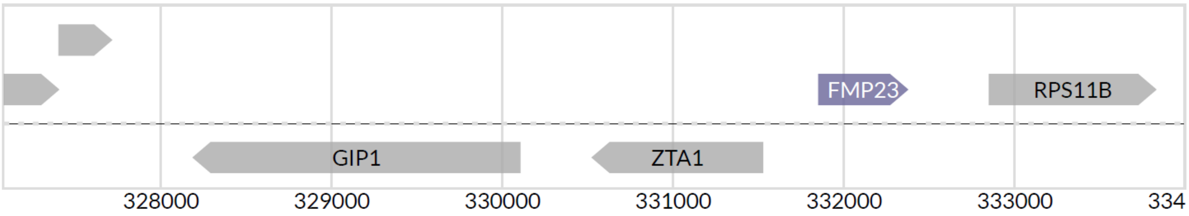

Fig 4. The region on chromosome II in *S. cerevisiae* that contains the FMP23, ZTA1 and GIP1 genes (from the *Saccharomyces* Genome Database).

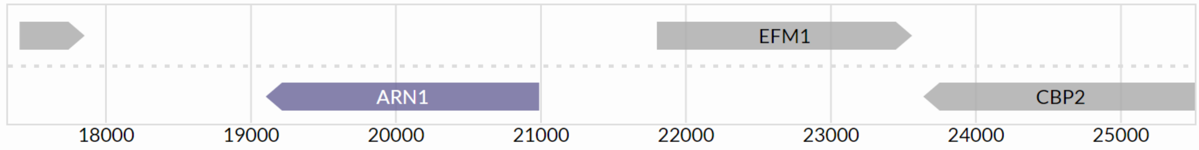

Fig 5. The region on chromosome VIII in *S. cerevisiae* that contains the ARN1 gene (from the *Saccharomyces* Genome Database).

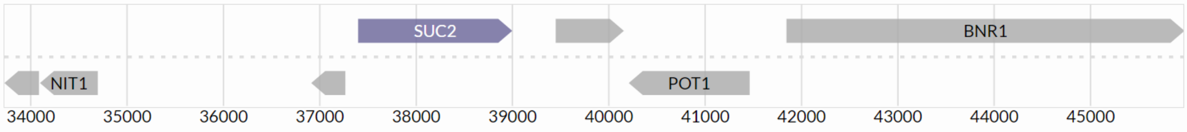

Fig 6. The region on chromosome IX in *S. cerevisiae* that contains the SUC2 gene, a homolog of the INU1 gene (from the *Saccharomyces* Genome Database).

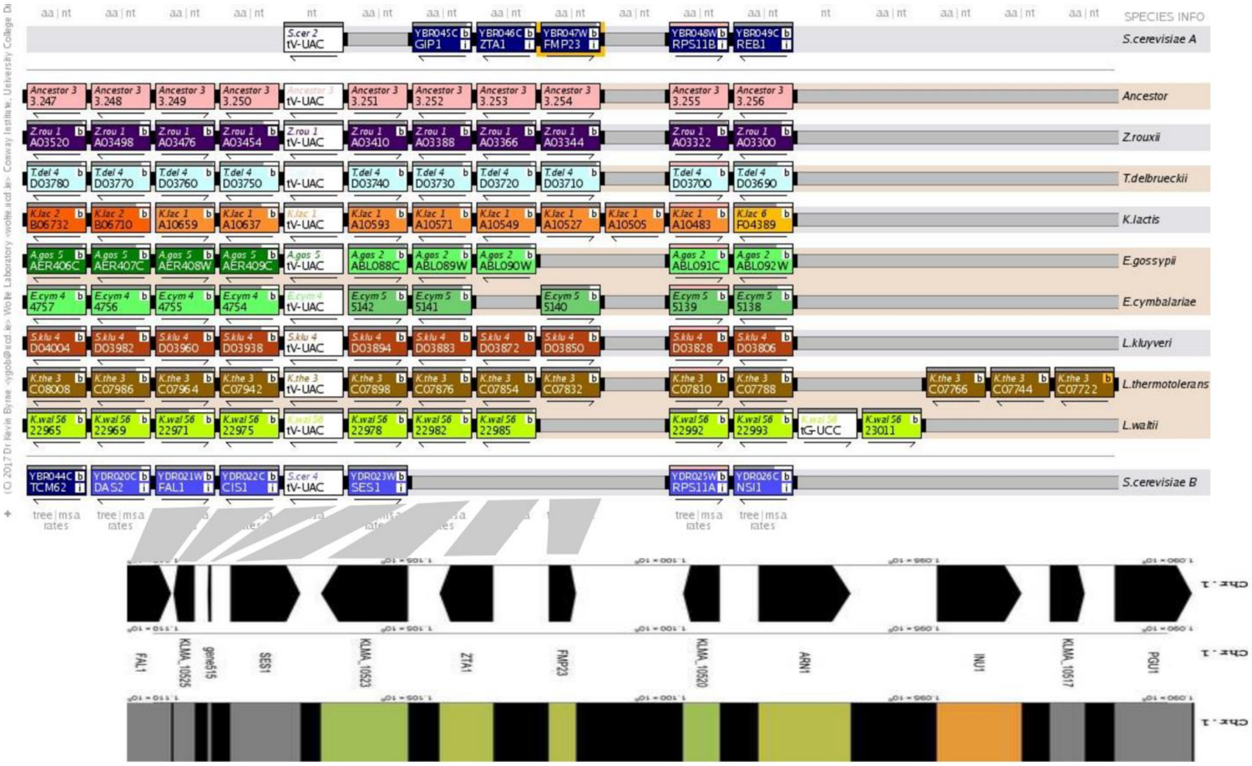

Fig 7. The region that contains the FMP23, ZTA1 and GIP1 genes mapped to the same region in the Yeast Gene Order Browser. GIP1, ZTA1 and FMP23 are conserved in a region of synteny in pre-genome duplication yeasts, along with FAL1, CIS1, the tRNA Gene515 (tV-UAC), and SES1.

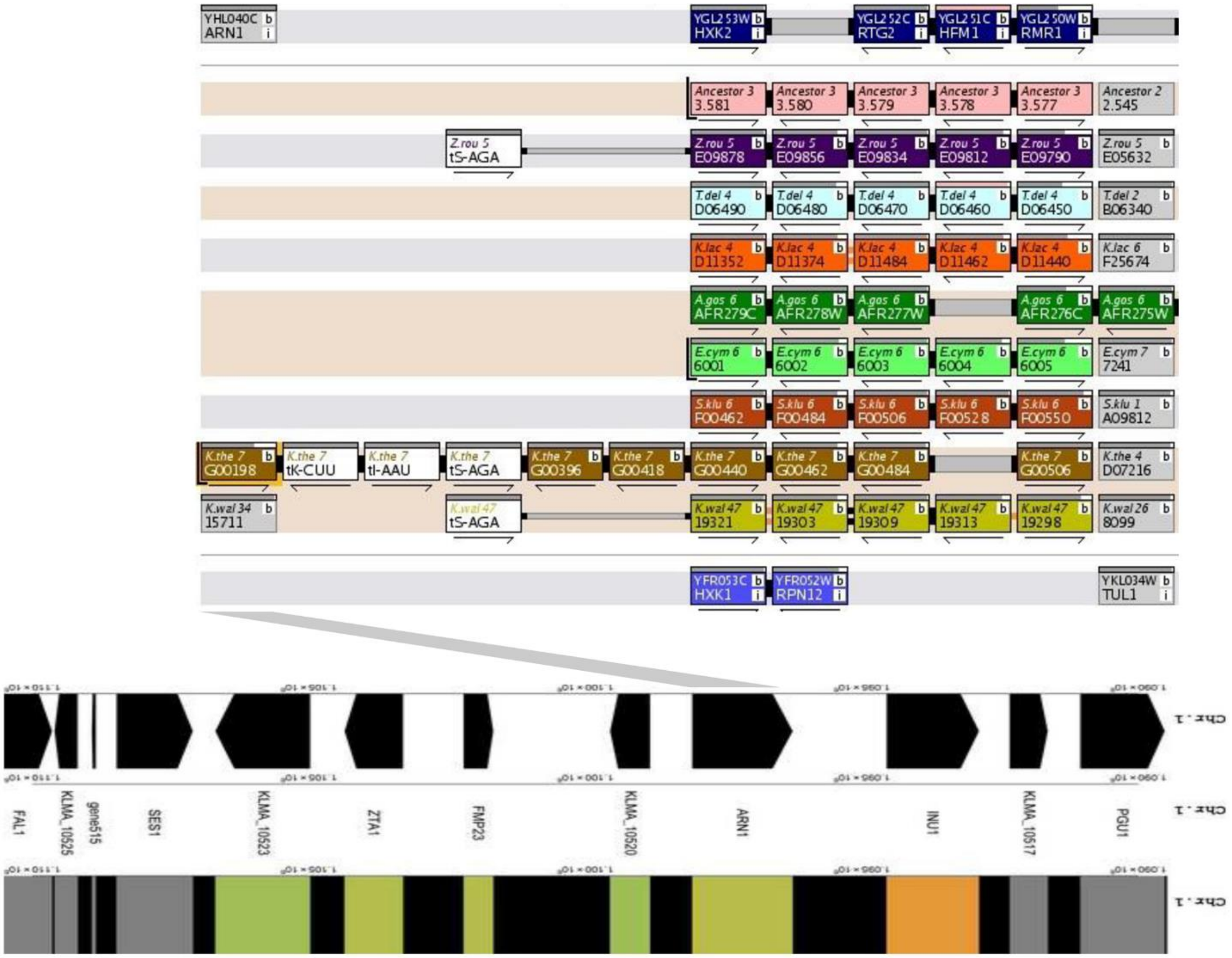

Fig 8. ARN1 is conserved in *Lanchaea thermotolerans* (*K. thermotolerans*), *L. waltii* (*K. waltii*) and *S. cerevisiae*, but absent from *K. lactis*, *L. kluyveri* (*S. kluyveri*) and other pre-genome duplication yeasts in the Yeast Gene Order Browser. The putative ARN1 homolog in *L. thermotolerans*, GOO198, is at the edge of a syntenic region. The INU1 gene does not seem to share synteny with the adjacent genes.

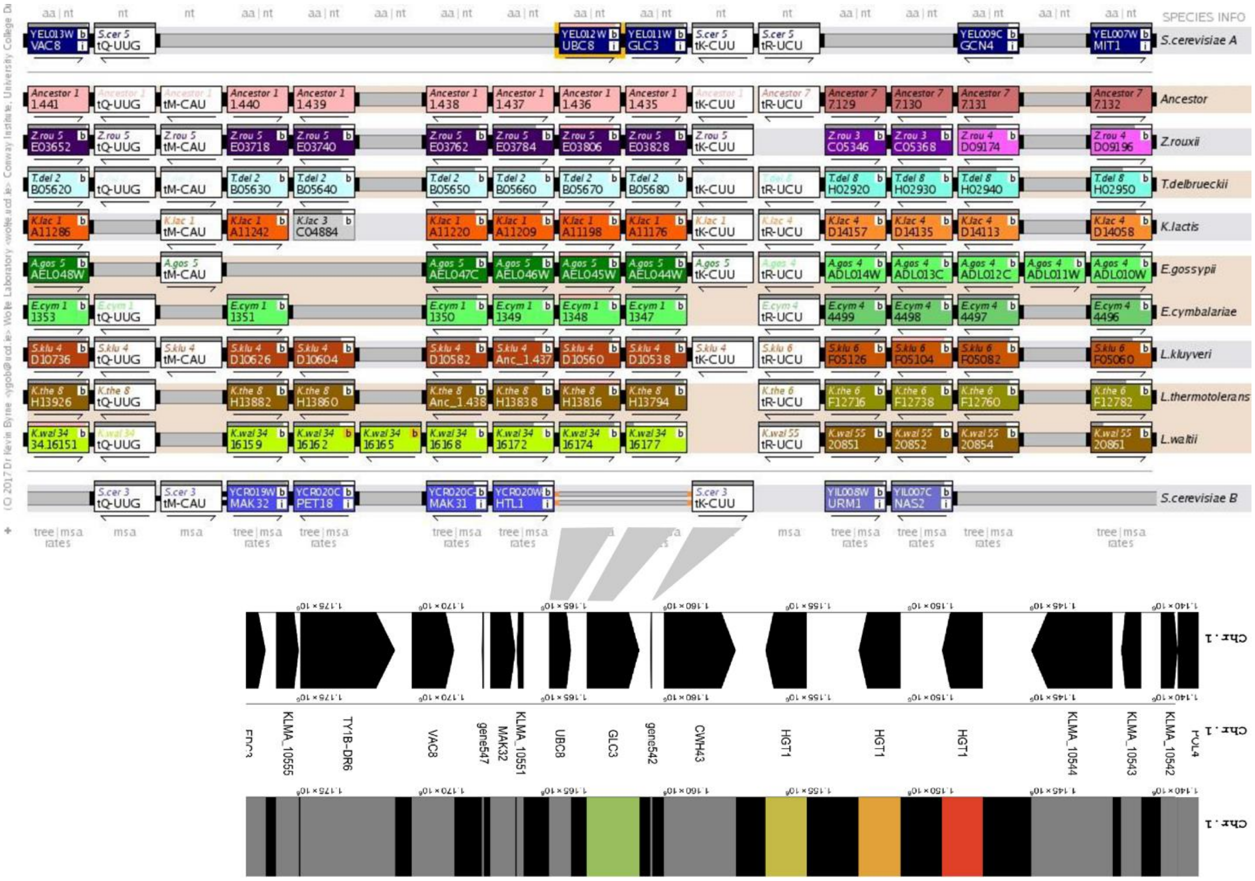

Fig 9. The region that contains the three copies of putative HGT1 gene homolog mapped to the same region in the Yeast Gene Order Browser.

Transcript level			Fold change (Xyl/Glc)		
	Total			Log2(FC)	FC
	1			-10	0.000977
	2..8			-8	0.003906
	7.82			-6	0.015625
	21.8			-4	0.0625
	61.1			-2	0.25
	171			0	1
	477			2	4
	1330			4	16
	3730			6	64
	10400			8	256
	29200			10	1024

Fig 10. Colour keys for gene expression maps. Left: Gene expression level as determined by CuffDiff from RNA-seq data. Right: Differential gene expression as a fold change of transcript level (xylose/glucose) as determined by CuffDiff.

Table 1. Transcript levels and fold changes for three gene clusters.

Gene ID	Most similar gene (UniProt)	Locus	Expr(GLC)	Expr(XYL)	log2FC	FC	p	q	significant
Cluster 1									
gene508	INU1	AP012213.1:1092882-1094553	127	11472	6.50	90.4	0.00005	0.0010	yes
gene509	ARN1	AP012213.1:1096251-1098069	6.8	55.7	3.03	8.2	0.00005	0.0010	yes
gene510	KLMA_10520	AP012213.1:1098831-1099554	71.4	218	1.61	3.0	0.0008	0.0107	yes
gene511	FMP23	AP012213.1:1101665-1102202	8.8	57.8	2.72	6.6	0.0001	0.0019	yes
gene512	ZTA1	AP012213.1:1103296-1104355	84.7	509	2.59	6.0	0.00005	0.0010	yes
gene513	KLMA_10523	AP012213.1:1104976-1106692	5.8	18.6	1.68	3.2	0.0009	0.0119	yes
Cluster 2									
gene538	HGT1	AP012213.1:1147865-1149536	7.5	6307	9.72	843	0.00005	0.0010	yes
gene539	HGT1	AP012213.1:1151229-1152945	4.8	293	5.93	61.0	0.00005	0.0010	yes
gene540	HGT1	AP012213.1:1155074-1156763	16.1	199	3.63	12.4	0.00005	0.0010	yes
gene541	CWH43	AP012213.1:1157967-1160907	140.1	97.2	-0.53	0.69	0.1647	0.4433	no
gene542	KLMA_R121	AP012213.1:1161388-1161463	0	0.0	NA	NA	NA	NA	NA
gene543	GLC3	AP012213.1:1161909-1164063	111.6	261	1.22	2.3	0.00145	0.0176	yes
Cluster 3									
gene4660	FOX2	AP012219.1:875894-878696	4.8	1356	8.15	285	0.00005	0.0010	yes
gene4661	KTR1	AP012219.1:879678-880938	16.0	8.2	-0.96	0.5	0.06935	0.2673	no
gene4662	KLMA_70428	AP012219.1:882326-887228	1.3	6.3	2.24	4.7	0.00025	0.0040	yes
gene4663	ICL2	AP012219.1:889129-890848	10.3	322	4.96	31.2	0.00005	0.0010	yes
gene4664	KLMA_70430	AP012219.1:891903-893043	6.7	21.6	1.68	3.2	0.00235	0.0250	yes
gene4665	KLMA_70431	AP012219.1:894291-895644	73.0	81.0	0.15	1.1	0.72785	0.8844	no
Cluster 3 neighbouring region									
gene4677	PDH1	AP012219.1:914330-915917	24.0	813	5.08	33.8	0.00005	0.0010	yes
gene4678	CIT3	AP012219.1:916923-918348	3.0	900	8.24	302	0.00005	0.0010	yes
