# Supplementary material for: Gene regulation in Kluyveromyces marxianus in the context of chromosomes
Source: PLoS One. 2018 Jan 18;13(1):e0190913. doi: 10.1371/journal.pone.0190913 (PMC5773181; doi:10.1371/journal.pone.0190913)
Supplement: S1 Stats Report — (PDF) [file pone.0190913.s002.pdf]

Discussion of Rationale in Statistical Methods

Sean van der Merwe, The Department of Mathematical Statistics and Actuarial Science, The University of the Free State

In hypothesis testing we begin by assuming that any pattern observed in the data has occurred by chance alone, and that should the experiment be repeated, it probably would not produce the same patterns again. We then pick a test statistic that we can use to evaluate the probability of the observed patterns under this assumption. Should the probability be very low then this suggests that our patterns did not occur by chance alone and may recur. The more hypothesis tests that are performed, the more likely it is to observe extreme patterns by chance alone, and the less reliable the hypothesis testing procedure becomes. Thus, the ideal is to conduct as few hypothesis tests per experiment as possible. In some cases, it is important to do many hypothesis tests on the same sample. In such cases we use a multiple testing adjustment to reduce the number of false positives to an acceptable level. However, such adjustments are crude approximations. It is always better to do a global test.

In the case of this experiment, a global test can be constructed to evaluate the major patterns in one calculation. We assume that the total number in each category is fixed, but where they occur is random. We then calculate a non-parametric estimate of the probability of observing patterns at least as extreme as those observed, under our assumption, also known as a p-

value. Since the value that I calculated is very small (1.5×10^{-4}), it provides evidence against the assumption of random occurrences and suggests that the patterns are not random.

The procedure for obtaining this p-value was described in the text under Materials and Methods, “Enrichment for clusters of concordantly transcribed genes”.
